# Coexistent gouty and infectious tenosynovitis in the hand: a case report and narrative review of comparable cases

**DOI:** 10.1080/23320885.2025.2545199

**Published:** 2025-08-09

**Authors:** Mohammad Nouri, Malak Alsaif, Abdulaziz Alnufaei, Turki Alhassan

**Affiliations:** a Division of Plastic Surgery, Department of Surgery, King Abdulaziz Medical City, Ministry of National Guard Health Affairs (MNGHA), Riyadh, Saudi Arabia; b King Abdullah International Medical Research Center (KAIMRC), Riyadh, Saudi Arabia; c College of Medicine, King Saud bin Abdulaziz University for Health Sciences, Riyadh, Saudi Arabia

**Keywords:** Gouty tenosynovitis, infectious tenosynovitis, digital amputation, tophus

## Abstract

Although less commonly in the hand, gouty tenosynovitis may present with symptoms resembling infection. Only a few case reports document such presentations, and reports of coexisting infection and gouty tenosynovitis are even more uncommon. A 32-year-old male with polyarticular tophaceous gout, noncompliant with medications, presented with a one-day history of right index finger swelling and redness. Investigations were suggestive of infectiousious tophus. Despite broad-spectrum antibiotics and rheumatologic interventions (colchicine, allopurinol, and corticosteroids), his condition deteriorated. Multiple incisions and drainages were performed without improvement. Persistent infection, confirmed to be methicillin-resistant Staphylococcus aureus (MRSA), complicated the underlying gouty inflammation. Standard therapies for infective tenosynovitis did not yield clinical resolution, presumably due to ongoing crystal-induced inflammation and compromised tissue. Ultimately, finger amputation was performed to control disease progression after all other salvage options failed. This case underscores the aggressive and destructive potential of gout when complicated by infection. Normal or relatively low serum uric acid levels do not exclude gout, and synovial fluid crystal analysis can be pivotal. Coexisting infection and gouty tenosynovitis in the hand can lead to severe tissue damage if misdiagnosed or inadequately treated. A high index of suspicion, multidisciplinary collaboration, and timely surgical intervention are crucial in preventing further morbidity. This case demonstrates that amputation may be necessary when infection remains unresponsive to standard treatments, emphasizing the importance of early diagnosis and aggressive management.

## Introduction

Gout is a metabolic disorder caused by the deposition of monosodium urate monohydrate crystals in the periarticular soft tissues [1]. Gout affects roughly 1–2% of adults worldwide, whereas pyogenic (infectious) flexor tenosynovitis accounts for only about 2.5–9.5% of all hand-infection cases [2,3]. Gout is clinically diagnosed and is typically marked by redness and swelling, most often involving the first metatarsophalangeal joint [4]. The most important risk factor for developing gout is hyperuricemia [5]. Factors contributing to hyperuricemia include metabolic syndrome, chronic kidney disease, and certain medications such as cyclosporine and diuretics [6]. Environmental exposures, including purine-rich diets and sugar-sweetened beverages, also increase serum urate levels and the risk of gout [7]. In addition, genetics plays a role, with 55 identified loci associated with the risk of gout [8]. Although the hand is less commonly affected, it may become involved in the later stages of the disease when serum urate levels are very high [9]. Gouty tenosynovitis of the hand is already documented, and at least one report has described its coexistence with infection. Our case sharpens the focus on the diagnostic complexity of infected tophaceous gout in the hand. Through a targeted narrative review, we distill recurring clinical patterns, common diagnostic traps, and pragmatic treatment strategies.

## Case presentation

We describe a 32-year-old male with polyarticular tophaceous gout affecting multiple joints who was non-compliant with his medications. He presented to the emergency department with a one-day history of right-index-finger swelling and redness.

**Figure 1.**
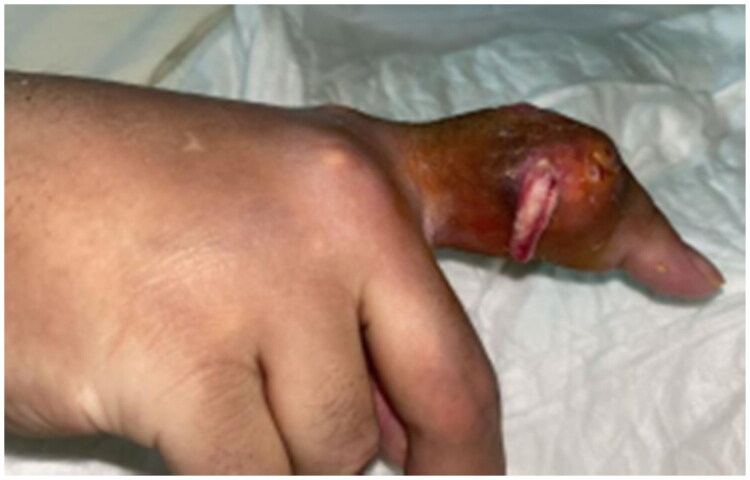
Patient condition upon presentation showing right index finger swelling and redness with extensive involvement of soft tissue.

On examination, he was vitally stable and afebrile. A large, ulcerated tophus was noted at the proximal interphalangeal joint (PIPJ) of the right index finger, with extensive soft-tissue involvement, swelling, erythema, and frank purulent discharge. Neurovascular examination was normal (Figure 1). Laboratory investigations showed a normal white blood cell count, a C-reactive protein (CRP) level of 14 mg/L, an erythrocyte sedimentation rate (ESR) of 26 mm/hour, a serum urate level of 5.7 mg/dL, and normal renal function (Table 1). *Gout was suspected clinically because of the visible tophus and chronic polyarticular disease, and this suspicion was later confirmed histologically.*

**Table 1.** Initial laboratory results.

WBC (×10⁹/L)	CRP (mg/L)	ESR (mm h⁻¹)	Serum urate (mg/dL)	eGFR (mL min⁻¹ 1.73 m⁻²)
6.2	14	26	5.7	98

**Figure 2.**
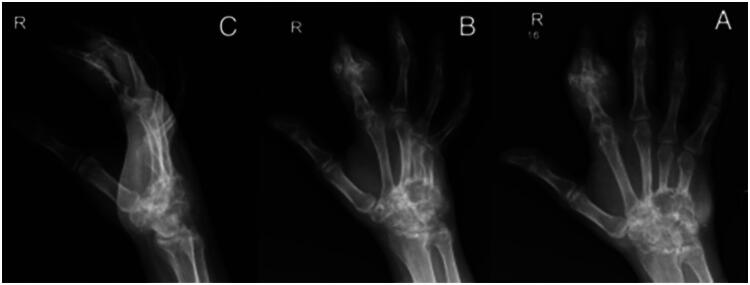
Left hand radiograph: (A) posteroanterior, (B) oblique, and (C) Lateral view. Shows significant left index finger soft tissue involvement with erosive changes and features of osteomyelitis.

Radiographs revealed diffuse soft-tissue swelling and features suggestive of osteomyelitis (Figure 2). *MRI was deferred because surgical exploration was imminent; DECT was unavailable at our centre.*

The patient was admitted with a diagnosis of infected tophi. *Empiric intravenous vancomycin (1 g q12 h) and cefepime (2 g q8 h) were started, and therapy was later de-escalated to oral trimethoprim–sulfamethoxazole after cultures confirmed methicillin-resistant Staphylococcus aureus (MRSA) sensitive to that agent.* The rheumatology team advised prednisolone 20 mg orally once daily in addition to his home colchicine 0.5 mg twice daily and allopurinol 400 mg once daily.

**Figure 3.**
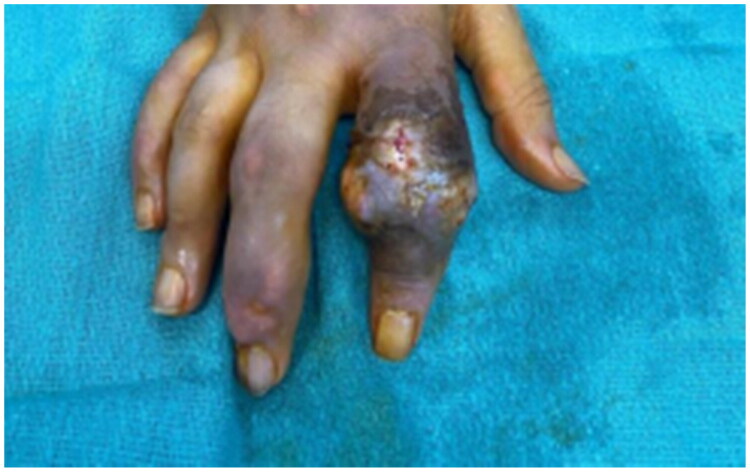
Index finger showing persistent redness and swelling after course of antibiotics and multiple incision and drainage.

Despite these interventions, his condition did not improve. On the seventh day of admission, he underwent incision and drainage with povidone-soaked gauze dressings applied three times daily (Figure 3). *Intra-operatively we noted opalescent, liquid tophus; a sample was sent for crystal analysis, confirming monosodium-urate crystals under polarised light.* Nevertheless, he continued to show no improvement, prompting amputation of the finger after full counselling when all salvageable options had been exhausted.

**Figure 4.**
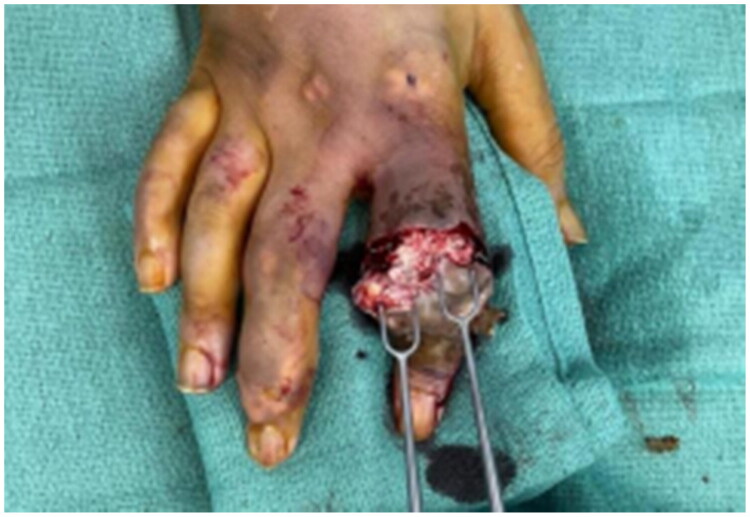
Post incision shows extensive tophaceous infiltration with extensive involvement of soft tissue.

**Figure 5.**
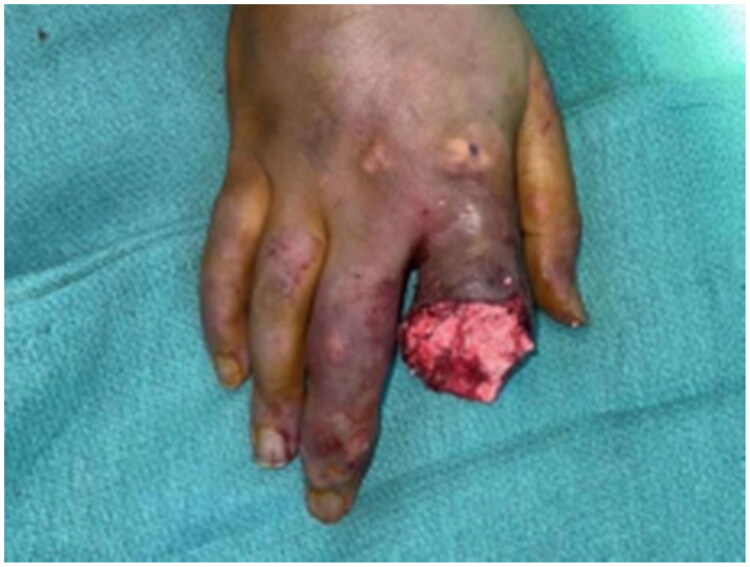
Post amputation at the PIPJ level.

**Figure 6.**
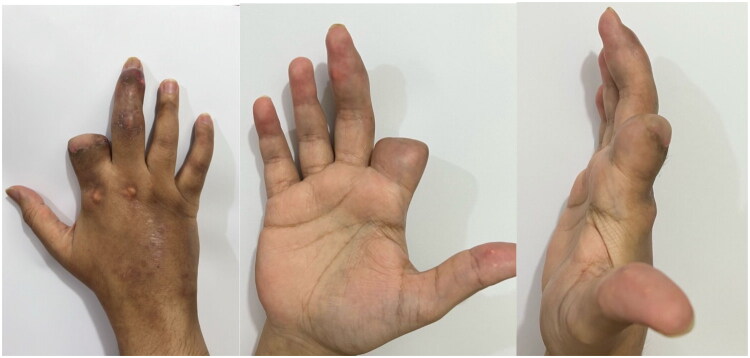
Dorsal, volar and lateral views showing healed post-amputation wound.

Under an infraclavicular block an incision was made using a volar skin flap, and amputation proceeded dorsally up to the PIPJ level using a bone cutter (Figure 4). Neurovascular bundles were cauterised bilaterally, and the nerve was transected at the PIPJ level (Figure 5). The skin flap was then transposed volarly over the proximal phalanx. The patient tolerated the procedure well and was discharged on *trimethoprim–sulfamethoxazole twice daily to complete six weeks of antibiotics*. On follow-up, the wound had healed with no evidence of infection or gout relapse (Figure 6).

## Discussion

Flexor-tendon tenosynovitis (FTS) of the hand is usually caused by trauma or bacterial infection, and less commonly by inflammatory diseases such as gout or rheumatoid arthritis [10]. Although FTS of gouty origin is uncommon, it should still be kept in mind when serum urate levels are elevated [9] or when patients show atypical findings resistant to standard antibacterial treatments. We identified 20 analogous episodes of gout-related flexor-sheath or tendon-sheath disease of the hand and wrist reported between 1995 and 2024 (Table 2).

**Table 2.** Published episodes of gout-related flexor-tendon or tendon-sheath disease of the hand and wrist (1985–2024).

Author(s) / Year	Site(s) of involvement	Clinical presentation	Key diagnostics	Treatment	Outcome
Lee DY, et al. / 2023 [11]	Right hand and forearm; flexor and extensor tendon sheaths; carpal tunnel	82-year-old male with history of gout presented with progressive pain, swelling, erythema, and signs of systemic inflammatory response, complicated by compartment syndrome and myonecrosis.	MRI: extensive tenosynovitis, cellulitis. Intra-op: diffuse tenosynovitis, tophi with pus-like discharge. Culture: negative.	Urgent fasciotomy of hand and forearm, serial debridement, staged skin grafting. Medical: colchicine, NSAIDs.	Wound fully closed by 3rd postoperative week; discharged after 6 weeks. Successful limb salvage.
Grilliot J, et al. / 2022 [12]	Left small finger flexor tendon sheath (A1 pulley)	58-year-old male with no history of gout presented with 5 days of atraumatic pain, swelling, and erythema mimicking pyogenic flexor tenosynovitis (positive Kanavel’s signs, fever, elevated ESR/CRP).	Labs: elevated ESR/CRP. Intra-op: copious chalky, sedimented material. Crystal analysis: MSU crystals+. Culture: negative.	Urgent surgical irrigation and debridement. Post-op: ibuprofen, oral corticosteroids.	Rapid improvement. At 1 year, no pain and full function, satisfied with outcome.
Patel A, et al. / 2022 [13]	Left middle finger flexor digitorum profundus (FDP) and superficialis (FDS) tendons	51-year-old male with history of mild gout experienced sudden ‘pop’ in finger, followed by swelling and loss of flexion.	Intra-op: extensive tophaceous and liquefactive gouty deposits, complete rupture of FDP, near-complete rupture of FDS. Histopathology: confirmed gout.	Stage 1: Debridement, washout, carpal tunnel release, silicone rod placement. Medical: colchicine. Stage 2 (3 months later): Palmaris longus tendon graft.	Full range of motion, including DIPJ, and resolution of scar hypersensitivity at 6 weeks post-reconstruction.
Lau L, Kwan K / 2021 [14]	Right thumb flexor pollicis longus (FPL) tendon at the wrist	46-year-old male with no prior history of gout presented with locked thumb (inability to extend IPJ), mimicking severe trigger thumb.	Intra-op: chalky mass infiltrating FPL tendon proximal to carpal tunnel after A1 pulley release failed to resolve locking. Histopathology: confirmed gouty tophi.	Surgical release of A1 pulley, extended to wrist for excision/debridement of tophaceous mass from FPL tendon.	Full passive extension of IPJ achieved intra-operatively. Good functional outcome.
Prescher H, et al. / 2021 [15]	Right middle finger flexor tendon at PIP joint	32-year-old male with history of podagra presented with 5 days of progressive swelling, pain, erythema, and loss of motion mimicking infection.	Labs: elevated uric acid. Intra-op: gouty tophus with extensive infiltration of flexor tendon. Pathology: urate crystals.	Surgical exploration, tenosynovectomy, and flexor tendon release.	Not specified, but surgery was performed to prevent permanent damage.
Meyer zu Reckendorf G, Dahmam A / 2020 [16]	Small finger FDP and FDS tendons at Camper’s chiasm	37-year-old male with history of gout presented with triggering of small finger.	Intra-op: gouty infiltration along length of FDP tendon, fusion of FDP/FDS. Histopathology: confirmed gouty tophus.	Exploratory surgery, tenolysis, A1 pulley release, excision of gouty material, and separation of fused tendons.	Regained full function of the affected finger postoperatively with no recurrence.
Doucet 2020 [17]	Left little-finger flexor sheath (A1/A2 pulleys)	“Trigger finger” with restricted flexion	Not reported	Excision of tophi + pulley release	Full ROM restored
Kim Y, et al. / 2019 [18]	Right middle finger flexor tendon	Patient with hyperuricemia presented with sudden inability to extend the right middle finger (flexion contracture), mimicking trigger finger or locking of the MP joint.	Intra-op: flexor tendon with deposited chalky white substance. Histology: confirmed gout.	Tenosynovectomy and removal of chalky substance.	Recovered nearly to a full range of motion of the affected digits.
Akram A, et al. / 2016 [10]	Left index finger flexor sheath	54-year-old man presented with purulent-appearing swelling mimicking infective flexor tenosynovitis.	Intra-op: purulent fluid. Culture: Staphylococcus aureus+. Crystal analysis: MSU crystals+.	Multiple surgical washouts for infection. Medical: prolonged antibiotics, colchicine, and allopurinol for gout.	Resolved with combined therapy.
Lemrhari Y, El Bouchti I / 2016 [19]	Left index finger flexor superficialis tendon	68-year-old woman with RA, diabetes, and CKD presented with recurrent episodes of tenosynovitis and a hard swelling on the volar aspect of the finger.	US: "snow storm appearance". Labs: elevated ESR, CRP, uric acid. Histology: confirmed gouty deposits.	Initial trial of antibiotics (no improvement). Surgical removal of gouty deposit. Medical: Anakinra, Febuxostat.	Regained normal active motion after physiotherapy.
Kumar N, et al. / 2016 [20]	Left middle finger FDS tendon in forearm	45-year-old man with no history of gout presented with inability to extend middle finger and a growing mass on volar forearm, mimicking TB or neoplasm.	MRI: mass within FDS tendon. Intra-op: whitish chalky infiltration of FDS tendon. Histopathology: confirmed tophaceous gout. Labs: elevated uric acid (8.6 mg/dL).	Synovectomy and excision of the infiltrated FDS tendon.	Not specified, but surgery relieved the contracture.
Coombs PR, et al. / 2006 [21]	Left middle finger FDP and FDS tendons	63-year-old man with no history of gout presented with 12 months of gradual inability to flex his middle finger and mild swelling.	US: atypical tenosynovitis with increased tendon echogenicity, heterogeneity, and attenuation. Intra-op: chalky deposits. Histology/Microscopy: confirmed gouty tophus, MSU crystals+. Labs: elevated uric acid (0.60 mmol/L).	Initial NSAIDs (no improvement). Surgical exploration, tenosynovectomy, and A1 pulley release (two separate operations).	Articular symptoms improved; receiving aggressive physical therapy.
Yoshihara 2005 [22]	Right ring-finger flexor sheath	Painful swelling, erythema; mimicked pyogenic flexor-tenosynovitis	**Not reported – intra-operative histology showed monosodium-urate crystals; no bacteria mentioned**	Tenosynovectomy → colchicine & urate-lowering therapy	Recurrence two years later; managed medically
Aslam N, et al. / 2004 [23]	Left index finger flexor sheath (A1 pulley to distal phalanx)	44-year-old female with no history of gout presented with an acutely swollen, painful, erythematous finger mimicking infection.	US: tenosynovitis with loculated fluid. US-guided aspiration: turbid fluid with MSU crystals+. Culture: negative. Labs: normal urate and inflammatory markers.	Medical management with colchicine, followed by a COX-2 inhibitor.	Rapid and marked improvement within 24 h. At 3 months, a pain-free, fully functional finger.
Weniger FG, et al. / 2003 [24]	Left index finger flexor sheath	54-year-old man with several-year history of gout presented with painless mobile masses and inability to flex index finger normally.	Labs: elevated uric acid (10.0 mg/dL). Intra-op: classic tophi with destruction of flexor sheath and involvement of FDS tendon.	Surgical exploration and debridement of tophi.	Good functional result.
Weniger FG, et al. / 2003 [24]	Left middle finger flexor sheath	72-year-old man with 30-year history of gout presented with multiple painful nodules on volar and dorsal finger, with marked limitation of motion.	MRI: showed tophaceous involvement. Intra-op: tophi with destruction of flexor sheath and involvement of FDS tendon.	Surgical exploration and debridement of tophi.	Good functional result.
Weniger FG, et al. / 2003 [24]	Right small finger flexor sheath	67-year-old man with 7-year history of gout presented with a painful, enlarging mass on the volar finger, initially diagnosed as a ganglion cyst.	Intra-op: tophi with destruction of flexor sheath and involvement of FDS tendon.	Surgical exploration and debridement of tophi.	Good functional result.
Schuind 2003 – Case 1 [25]	Left index/middle/ring flexor sheaths + carpal tunnel	Dysesthesias, thumb ankylosis, multiple digital tophi	Not reported	Excision of tophi; tenolysis of flexor-pollicis-longus; median-nerve neurolysis	Dysesthesias resolved; ROM regained
Schuind 2003 – Case 2 [25]	Left flexor-pollicis-longus	Trigger-thumb–like locking	Not reported	FPL reconstruction using palmaris-longus graft	Full active ROM post-op
Abrahamsson SO / 1987 [26]	Right middle finger flexor tendon sheath	74-year-old man with history of podagra presented with 2 days of increasing pain, swelling, and reduced motion in finger, with fever, mimicking infection.	Labs: elevated ESR, serum urate. Intra-op: gouty deposits penetrating flexor tendon sheath. Specimen fixed in ethanol for crystal preservation.	Initial drainage for suspected infection. Reoperation with tenosynovectomy and excision of intratendinous tophi. Medical: indomethacin, allopurinol.	Almost normal active range of movement at 6 months.
Moore 1985 – Case 1 [27]	Wrist / carpal tunnel	Thenar wasting, nocturnal wrist pain; later extensor-pollicis-longus (EPL) rupture	Not reported (no infection described)	Median-nerve neurolysis; tophus excision; later EIP→EPL tendon transfer	Symptom relief and return of thenar bulk; functional EPL after transfer
Moore 1985 – Case 2 [27]	Left ring-finger extensor mechanism	Nodular masses limiting motion	Not reported	Tenolysis + synovectomy of extensor mechanism	65° PIP arc; patient satisfied at 2 yrs
Moore 1985 – Case 3 [27]	Right small-finger flexor sheath	Post-trauma swelling, limited motion; misdiagnosed initially	Not reported	Flexor tenolysis + tophus excision; colchicine & allopurinol	Lacked only 5° PIP and 15° DIP flexion at 6 yrs; no flares

Table 2 consolidates these 20 episodes. All presented with clinical hallmarks of septic FTS—acute swelling, erythema, pain and, in several reports, true Kanavel’s signs—yet only two (Akram 2016 and the current case) yielded a positive bacterial culture, both for *Staphylococcus aureus*. In every other instance the aspirate was culture-negative, and the diagnosis pivoted on intra-operative discovery of chalky material followed by crystal confirmation. The downstream spectrum was wide: “trigger-finger”–like locking [23,14], compartment syndrome with incipient myonecrosis [11], and spontaneous flexor-tendon rupture requiring staged reconstruction [13]. Most patients underwent at least one débridement or tenosynovectomy; eight required repeat washouts or staged procedures, whereas four improved with medical therapy alone once infection was excluded. Outcomes were generally excellent when crystals were identified early and urate-lowering therapy accompanied surgery; the single amputation occurred in our patient, underscoring the destructive synergy of true infection and tophaceous gout.

Gout most frequently involves the first metatarsophalangeal joint, but when it affects the hand it typically appears in the chronic phase of the disease. Common manifestations include subcutaneous tophi at the finger joints [28]. Yoshihara et al. [22] reported a 50-year-old man who initially presented with symptoms mimicking infectious FTS; histopathology later confirmed gouty tenosynovitis. Arthrocentesis was considered but not performed pre-operatively because the ulcerated tophus was draining freely and urgent surgical débridement was planned; instead, purulent material obtained intra-operatively was sent for both culture and crystal analysis.

Gouty tenosynovitis can clinically imitate septic tenosynovitis, often producing purulent or pus-like fluid. However, repeated negative cultures and the presence of monosodium-urate crystals differentiate gout from true infection [10,23,26]. Misdiagnosis is common: in 18⁄20 published episodes—including ours—the initial treatment targeted infection alone, delaying definitive management. It is also crucial to recognise that normal serum urate does not exclude gout; several patients in Table 2 had normal levels at presentation [14,23,20].

When clinical and laboratory findings are inconclusive, advanced imaging—such as ultrasound, MRI, or computed tomography—can help identify urate-crystal deposits or tophaceous infiltration. Combined with synovial-fluid analysis, imaging aids in distinguishing atypical gout from a true bacterial process [27].

Without treatment, deposits may invade cartilage and subchondral bone, creating cavities and indentations [29]. Liquefaction of tophi or severe inflammation can produce fistulae, sinus tracts, or even compartment syndrome [11]. Chronic gouty inflammation compromises local tissues, increasing the risk of superimposed infection and necrosis.

While medical management and lifestyle modification remain the mainstay for most gout patients, about 5% do not respond to standard regimens [30]. According to Straub et al. [31], surgical intervention in tophaceous gout of the hand is indicated to restore function, control drainage and infection, relieve pain, decompress nerves, and alleviate mechanical obstruction caused by bulky tophi. Tripoli et al. [32] described three techniques: (1) tenosynovectomy for heavy tendon infiltration; (2) shaving procedures for skin infiltration with ulceration; and (3) complex excision of larger deposits to improve range of motion.

Collectively, the literature and our case underscore three practical points: (1) aspirate or biopsy any chalky or opalescent material and send it for crystal analysis; (2) initiate empirical antibiotics promptly but refine therapy once culture and crystal results return; and (3) do not delay surgical exploration when clinical deterioration persists, as irreversible tendon or soft-tissue loss can ensue.

We present a case of coexisting infection and gouty tenosynovitis in the hand, highlighting key diagnostic pitfalls. Despite intravenous antibiotics, gout medical management, and multiple sessions of incision and drainage, the patient’s symptoms progressively worsened. Ultimately, finger amputation was performed after all salvageable options were exhausted and with full patient counselling. Such an aggressive outcome emphasises the destructive potential of gout when compounded by infection.

## Conclusion

Gouty tenosynovitis in the hand, while not routinely encountered, can cause devastating tissue loss if misdiagnosed or under-diagnosed. Although gout is not usually listed among primary causes of hand tenosynovitis, it must remain in the differential—particularly in patients with elevated serum urate levels or symptoms that do not respond to antibiotics. As demonstrated by our case and analogous reports, its clinical picture can closely mimic infection, especially when purulent discharge is present. Importantly, normal serum urate does not exclude gout; synovial-fluid or tissue crystal analysis remains essential for definitive diagnosis. In any atypical flexor-sheath infection, aspiration or biopsy of chalky or opalescent material for gouty investigation, might improve overall prognosis by targeting the right diagnosis.

When less-invasive options such as incision-and-drainage or tenosynovectomy fail, more radical procedures—including amputation—may be unavoidable to halt disease progression and preserve overall function. Ultimately, a high index of suspicion and a multidisciplinary approach are vital for timely diagnosis and effective management.
